# A Comprehensive Analysis of COVID-19 Vaccine Discourse by Vaccine Brand on Twitter in Korea: Topic and Sentiment Analysis

**DOI:** 10.2196/42623

**Published:** 2023-01-31

**Authors:** Susan Park, Young-Kyoon Suh

**Affiliations:** 1 Institute of Health and Environment Seoul National University Seoul Republic of Korea; 2 Institute for Community Care and Health Equity Chung-Ang University Seoul Republic of Korea; 3 School of Computer Science and Engineering Kyungpook National University Daegu Republic of Korea; 4 Department of Data Convergence Computing Kyungpook National University Daegu Republic of Korea

**Keywords:** COVID-19, vaccine, vaccination, Pfizer, Moderna, AstraZeneca, Janssen, Novavax

## Abstract

**Background:**

The unprecedented speed of COVID-19 vaccine development and approval has raised public concern about its safety. However, studies on public discourses and opinions on social media focusing on adverse events (AEs) related to COVID-19 vaccine are rare.

**Objective:**

This study aimed to analyze Korean tweets about COVID-19 vaccines (Pfizer, Moderna, AstraZeneca, Janssen, and Novavax) after the vaccine rollout, explore the topics and sentiments of tweets regarding COVID-19 vaccines, and examine their changes over time. We also analyzed topics and sentiments focused on AEs related to vaccination using only tweets with terms about AEs.

**Methods:**

We devised a sophisticated methodology consisting of 5 steps: keyword search on Twitter, data collection, data preprocessing, data analysis, and result visualization. We used the Twitter Representational State Transfer application programming interface for data collection. A total of 1,659,158 tweets were collected from February 1, 2021, to March 31, 2022. Finally, 165,984 data points were analyzed after excluding retweets, news, official announcements, advertisements, duplicates, and tweets with <2 words. We applied a variety of preprocessing techniques that are suitable for the Korean language. We ran a suite of analyses using various Python packages, such as latent Dirichlet allocation, hierarchical latent Dirichlet allocation, and sentiment analysis.

**Results:**

The topics related to COVID-19 vaccines have a very large spectrum, including vaccine-related AEs, emotional reactions to vaccination, vaccine development and supply, and government vaccination policies. Among them, the top major topic was AEs related to COVID-19 vaccination. The AEs ranged from the adverse reactions listed in the safety profile (eg, myalgia, fever, fatigue, injection site pain, myocarditis or pericarditis, and thrombosis) to unlisted reactions (eg, irregular menstruation, changes in appetite and sleep, leukemia, and deaths). Our results showed a notable difference in the topics for each vaccine brand. The topics pertaining to the Pfizer vaccine mainly mentioned AEs. Negative public opinion has prevailed since the early stages of vaccination. In the sentiment analysis based on vaccine brand, the topics related to the Pfizer vaccine expressed the strongest negative sentiment.

**Conclusions:**

Considering the discrepancy between academic evidence and public opinions related to COVID-19 vaccination, the government should provide accurate information and education. Furthermore, our study suggests the need for management to correct the misinformation related to vaccine-related AEs, especially those affecting negative sentiments. This study provides valuable insights into the public discourses and opinions regarding COVID-19 vaccination.

## Introduction

### Background

Despite progress in reducing disease mortality and morbidity in regions with high vaccination rate, challenges remain owing to uncertainties from the recently identified variants of COVID-19 [1]. Moreover, because of the short duration of vaccine-induced immunity against SARS-CoV-2 and the uncertainties associated with the variants, follow-up or booster vaccinations may be required [2,3]. In this context, it may be necessary to consider how people respond to the continuing demand for vaccination and how the government can recommend vaccination to the public.

Understanding public opinion regarding COVID-19 vaccines is important for public health. Numerous studies have attempted to analyze topics and sentiments regarding COVID-19 vaccines using social media data, such as that from Twitter and Facebook [4-8]. However, these studies are mainly limited to data from 2020 to early 2021, which was the initial stage of vaccine development and rollout. In addition, the government’s responses to COVID-19, including vaccination policies, have changed over time. For example, the Korean government implemented a COVID-19 vaccine pass policy in December 2021 in response to the spread of Omicron variants. Changes in government policies can lead to changes in public reaction.

In addition, public opinion regarding COVID-19 vaccines may be closely related to concerns about adverse events (AEs). The unprecedented speed of messenger RNA vaccine development and approval has raised concerns that clinical trials were hastened and regulatory standards were relaxed [9]. Several recent topic modeling studies have reported concerns about AEs as a common major topic [10-13]. Moreover, most countries have used various vaccine brands (eg, Moderna, Pfizer, AstraZeneca, and Jassen), each with different use guidelines and safety and efficacy profiles [14,15]. However, there have been few in-depth studies on topics and sentiments focused on AEs, particularly over long periods after rollout.

Korea reported that 70% of its population had already been fully vaccinated within 8 months of the start of the vaccination drive on February 26, 2021 [16]. This result indicates that Korea reached the target vaccination rate approximately 2 months earlier than other countries that started vaccination earlier (eg, the United States, the United Kingdom, and France). As of May 31, 2022, the proportions of the Korean population with complete vaccination and an additional booster shot were 86.8% and 66.9%, respectively, making Korea one of the countries with the highest vaccination rates worldwide [16].

In this study, we explored the overall and brand-specific topics and sentiments related to COVID-19 vaccines in Korea after vaccine rollout. In addition, we examined their topic changes over time.

### Research Questions

For research purpose, we raised and answered the following research questions:

1. What topics have been discussed on Twitter regarding COVID-19 vaccines in Korea?
2. What are Twitter sentiments regarding the COVID-19 vaccines in Korea? Are they negative, positive, or neutral? Do these sentiments change over time?
3. What specific topics with respect to vaccine brand types, including Pfizer, Moderna, Jassen, and AstraZeneca, are discussed on Twitter in Korea? Are there any differences among these topics?
4. How about their sentiments? Do the sentiments change over time?
5. What are the specific topics on Twitter in Korea with terms related to AEs of COVID-19 vaccines?

### Related Works

We reviewed a rich body of existing literature on topics and sentiments related to COVID-19 vaccines using social media data (Multimedia Appendix 1 [4-8,10-13,17-30]). The major data source was Twitter (21 studies), and other sources were Reddit (2 studies), Facebook (1 study), and Weibo (1 study). The period of the collected literature was from the end of 2020 to the beginning of 2021. The average duration of the data collection was 5.14 months. The shortest data collection period was 8 days [17], and the longest data collection period was approximately 1 year [11]. Only 3 studies collected data for >10 months.

### Topics About the COVID-19 Vaccines

Prior studies on topic modeling have shown vaccine safety and efficacy, vaccine development, national vaccination policies, and vaccine supply to be major topics in a broad framework. The most commonly derived main topic was the concern about AEs, which was the main topic reported in 6 studies [4,6,10-13]. Previous topic modeling studies have shown that vaccine efficacy [8,11,17,18] and hesitancy [4,5,19] are the most common topics. Vaccine development and progress were the second-most discussed topics, discussed in 5 studies [8,11,18,20,21]. Certain people, such as Bill Gates and Kamala Harris, were mentioned as topic words [11,12]. National vaccination policies, such as mask-wearing practices and social distancing measures, were important public discourse [12,22,23]. In addition, trust in the government and medical institutions [12,17], misinformation about vaccines [12], and conspiracy theories [18] have been discussed as major topics. Several topic modeling studies have identified supply [4,24], distribution [6,11], and access to vaccines [6,7] as important topics. Some studies have determined vaccination priority as a major topic and discussed discourses regarding disagreements with the recommendations of the Center for Disease Control [11,12].

### Sentiments About the COVID-19 Vaccines

A total of 20 studies presented sentiments or emotions expressed on social media regarding COVID-19 vaccines. Most of these were analyzed based on positive, neutral, and negative feelings. Nine studies showed that positive sentiments prevailed over other sentiments (neutral and negative) [4,10,11,13,23,25-27,29]. Neutral emotions were dominant in 1 study [28] and negative sentiments prevailed in 3 studies [6,7,21]. Several previous studies have revealed that opinions vary according to the data collection period, region, and social media platforms. In particular, 1 study showed that the sentiment scores for COVID-19 vaccines were significantly different among 3 cities in Canada [24]. Two other studies revealed difference in sentiments by country and social media platform [19,20]. One study showed time-varying sentiments [30]. Approximately half (50%) of Twitter users in the United States expressed neutral sentiment toward the vaccines, and 40.6% of Weibo users in China indicated a positive opinion [20]. In Korea, the ratios of positive and negative sentiments of Twitter users before and after vaccination were similar. However, as the number of confirmed cases increased, the number of negative tweets also increased [19]. In total, 20% (4/20) of studies analyzed emotions in a more diverse manner. Monselise et al [7] analyzed 5 emotions: positive, such as joy and hopefulness, and negative, such as fear, sadness, and anger. The remaining 3 studies used the following 8 emotions: anger, fear, anticipation, trust, surprise, sadness, joy, and disgust [11,23,25].

## Methods

### Ethics Approval

This study was ethically approved by the KNU Institutional Review Board (KNU-2021-0118).

### Overview

**Figure 1:**
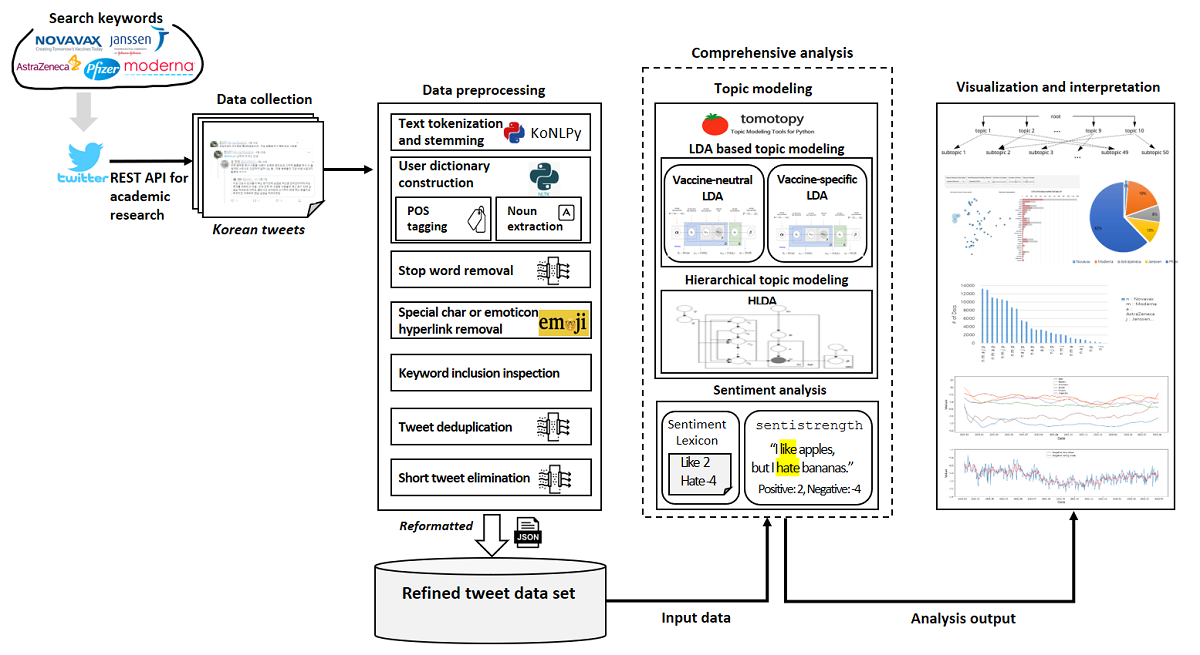
Overall methodology for COVID-19 vaccine discourse analysis on Twitter in Korea. This illustrates how our analysis was conducted from data collection to outcome visualization. API: application programming interface; HLDA: hierarchical latent Dirichlet allocation; LDA: latent Dirichlet allocation; POS: parts of speech.

This section briefly describes the methods used in this study. More technical details regarding the methods are provided in Multimedia Appendix 2. Figure 1 depicts the overall methodology for our analysis, including keyword search on Twitter, data collection, data preprocessing, topic modeling, sentiment analyses, and output visualization and interpretation.

### Data Collection and Preprocessing

We built a large corpus by collecting Korean tweets mentioning COVID-19 vaccine brands (eg, Pfizer, Moderna, AstraZeneca, Janssen, and Novavax), via academic research access [31] authorized by Twitter, posted from February 2021 to March 2022. We removed emoticons from the collected documents before subsequent preprocessing and then constructed an initial corpus with 1,689,158 tweets.

In turn, we preprocessed the initial corpus through a series of steps from stemming through short-tweet elimination. In particular, it was found that some of the tweets in the corpus were invalid for the study. There were 2 reasons for such invalidity. First, some of the tweets were retweets. Second, some tweets were not from the general public but from government offices (eg, Gyeongsangbuk-do Provincial Office), advertisements, news media companies (eg, MBC, KBS, and SBS broadcasting company), disaster alert bots (eg, dailycoronabot), and bots collecting play scripts of a character identical to a vaccine brand’s name (eg, Jassen). Multimedia Appendix 3 presents a complete list of these invalid accounts. Thus, invalid tweets had to be removed. Tweets containing keywords with different meanings from the given keywords were also excluded. Any included web links were removed, and various synonyms were replaced with common representative words in the retained tweets. As a sanity check, if the retained tweet was an empty string or contained >2 words, the tweet was discarded. Through rigorous preprocessing, 165,984 tweets were retained and used for this study. We then reformatted each preprocessed tweet into a JSON file, which was stored in MongoDB (MongoDB Inc).

### Analysis

We conducted a suite of analyses on the refined corpus with preprocessed tweets, such as latent Dirichlet allocation (LDA)-based topic modeling, hierarchical topic modeling, and sentiment analysis. First, the LDA analysis consisted of 2 phases. In the first phase, coarse-grained LDA analysis was run to determine the general trend, regardless of the vaccine brands. In the second phase, a fine-grained LDA analysis was conducted, focusing on each vaccine brand. To conduct these LDA analyses, a morpheme analysis was run and parts of speech (POS) tagged as common noun (NNG) and proper noun (NNP), denoting general and proper Korean nouns, were extracted from the analysis, and then, the specified vaccine brand names were removed to avoid affecting the analysis. A refined corpus consisting of the identified Korean nouns was embedded as term frequency–inverse document frequency (TF-IDF) for LDA analysis. In particular, we created vaccine-specific topics using pining topic modeling. Pining topic modeling allows to control word prior for each topic. The weight of the word “Pfizer” was set to 1.0 in topic 0, and the weight was set to 0.1 in the rest of the topics by following codes. Likewise, 10 times weight was given to the word “Moderna” in Topic 1, “AstraZeneca” in Topic 2, “Janssen” in Topic 3, “NovaVax” in Topic 4. This allowed the manipulation of topics to be placed at a specific topic number. To determine the optimal number of topics, we examined various indicators such as coherence [32], perplexity [33], and others [34-37] (Multimedia Appendix 4). We then determined that 50 is the appropriate number of topics based on these indicators. Topic labels justified by the validation process were assigned to the top 10 major topics [38]. The 3 coders labeled topics based on the topic words and original Twitter texts. Then, 2 tasks for the validation test were designed, namely, label intrusion (LI) and optimal label (OL), consisting of randomly selected Twitter text and 4 possible topic labels. For the LI task, 3 possible topic labels that were most associated with the Twitter text were chosen. At the same time, 1 topic was selected from other labels related to the remaining 9 topics. In the OL task, one was the highest probable label, and the others were chosen from the 9 unrelated topic labels. The 3 coders answered 10 questionnaires per topic, 5 each for the LI and OL tasks. The validity process measured the accuracy of the coders’ labels by confirming or refuting the assigned topic labels. The measured accuracy values for total, LI, and OL were 86.33%, 90.67%, and 82%, respectively, suggesting that our topic labels were valid.

Second, our hierarchical topic modeling analysis was conducted using the same TF-IDF. For this analysis, we built and trained a hierarchical LDA model 1000 times. During training, we were able to prevent the number of topics from drastically increasing by restricting new topic generation through an activated option (called freeze topics). Topic pruning was then applied to the trained model because too many topics made the interpretation challenging. To perform this pruning, the top *k* topics were selected at depth 1, based on the number of documents associated with each topic. For each topic, the top 3 subtopics were selected. That is, we checked if the number of documents belonging to a subtopic was greater than a threshold (ie, 330/165,984, 0.2% of the total documents in our data set), and that subtopic became a candidate for that selection. The candidates were sorted according to the order of their document counts and then the top 3 were selected. By doing so, a pruned and refined hierarchical LDA model was obtained. LDA and hierarchical topic modeling analyses were performed after embedding them as TF-IDF with Korean nouns.

Third, we performed sentiment analysis on the full corpus. For this analysis, SentiStrength (version 0.0.9) [39], a Python package that is one of the most popular programs, was used. The SentiStrength program uses sentences as basic units after translating the Korean text into English. This is because, by comparing the sentiment analysis results using other Korean lexicons, such as SentiStrength’s Korean lexicon and the Kunsan National University Korean Sentiment Dictionary [40], the results of sentiment analysis after translation into English showed the best performance. The results of sentiment score comparison between Korean and English are presented in Multimedia Appendix 5. To compute the sentiment score, we summed the positive and negative sentiment scores associated with the translated data (via getSentiment() in SentiStrength). Tweets with a sum of sentiments <0 were classified as negative, 0 as neutral, and >0 as positive.

Finally, to test the mean difference in sentiment scores for each vaccine brand, ANOVA and Tukey post hoc test were performed. Time trends were examined using an autoregression model to estimate the linear regression for time series data when the errors were autocorrelated. To analyze the structural changes in the model parameters, the Chow Test for Structural Breaks was performed using this procedure [41].

We implemented the methodology shown in Figure 1 in Python version 3.7.13. Various Python packages were used, such as emoji version 1.7.0, konlpy version 0.6.0, nltk version 3.7, numpy version 1.21.6, pandas version 1.3.5, pyLDAvis version 3.3.1, SentiStrength version 0.0.9, and tomotopy version 0.12.2. Statistical analyses were performed by SAS Studio (SAS Inc).

## Results

### Top 10 Topic Analysis on Korean Tweets Related to COVID-19 Vaccines

Table 1 shows the top 10 topics based on the proportion of Korean tweets about COVID-19 vaccines during the collection period. These results were obtained for research question 1. Among the top 10 topics, 6 subjects (topic numbers 3, 4, 17, 20, 32, and 40) were associated with adverse vaccination events and people’s feelings, accounting for 32.29% (53,596/165,984) of all Twitter posts.

The main words in topics 3, 17, and 40 were related to systemic reactions after vaccination, such as muscle pain, headache, fatigue, mild fever, and chills. Topic 20 was mainly related to local reactions, such as specific areas on the body around the injection site (left arm, shoulder, and armpit). Topic 32 revealed experiences and various feelings (eg, pain, worry, relief, and gratitude) regarding vaccination and AEs. The keywords of topic 26 were about AEs related to COVID-19 vaccination of family and friends.

**Table 1:** Top 10 topics about COVID-19 vaccines in the collected Twitter data in Korea (N=165,984).

Topics	Proportion, n (%)^a^	Topic words
Topic 17: systemic reaction after vaccination	15,193 (9.15)	Muscle pain, headache, momsal^b^, symptom, Tylenol, pain, progress, mild fever, energy, and chill
Topic 32: emotional reaction about vaccination	12,256 (7.38)	Worry, thank, booster shot, AE^c^, relief, suffering, cross vaccination, health, flu, and mind
Topic 20: injection site pain	8371 (5.04)	Pain, left arm, ache, muscle pain, progress, left Geumgangmakgi^d^, armpit, and muscle
Topic 26: concern about vaccination of intimate persons	8060 (4.86)	Mom, dad, friend, worry, little brother, AE, around, family, talking, and parents
Topic 4: news report about vaccine	6188 (3.73)	Press, AE, news, Giraegi^e^, problem, government, Korea, United States, report, and people
Topic 3; health condition after vaccination	5963 (3.59)	AE, head, mental, sick, all day, stunned, feeling, condition, and pain
Topic 21: vaccination day	5807 (3.50)	Friday, work, company, vacation, Saturday, weekend, Monday, school, Thursday, and booster shot
Topic 36: no-show vaccine reservation success story	5626 (3.39)	No-show vaccine, success, application, residual, hospital, alarm, neighborhood, Naver, ticketing, and waiting
Topic 40: taking analgesics to control pain and fever	5625 (3.39)	Tylenol, momsal, morning, muscle pain, head, chills, pain killers, headaches, taking, and sick
Topic 18: how to book a no-show vaccine	4725 (2.85)	Hospital, text, call, change, no-show vaccine, information, application, contact, date, and select

^a^This represents the number of tweets assigned to the topic with the highest probability because 1 tweet has >1 topic.

^b^The word “momsal” is a condition caused by extreme fatigue in which one’s body aches and suffers from exhaustion or fever.

^c^AE: adverse event.

^d^The word “Geumgangmakgi” is a traditional Korean taekwondo technique that features a defensive posture with arms raised.

^e^The word “Giraegi” is a combination of gija, the Korean word for journalists, and tsuraegi, the Korean word for trash.

Topics 18, 21, and 36 were related to vaccine access. The Korean government has recommended paid sick leaves in workplaces or official absence from schools on the day of vaccination. Thus, people preferred to get their vaccines on Thursdays or Fridays to take a break until the weekend (topic 21). No-show vaccines could be reserved on a first-come, first-served basis through a specific website (eg, Naver) or by phone call to designated clinics. Topics 36 and 18 pertained to web-based and phone reservations, respectively. Topic 4 concerned the news media and the governments of Korea and America. We also performed topic modeling by dividing data by quarterly period (data now shown). As a result, it was confirmed that the topic of vaccination changed over time, and the topic of AE prevailed in the later period compared with the initial period of vaccination.

### Hierarchical Topic Modeling Analysis

**Figure 2:**
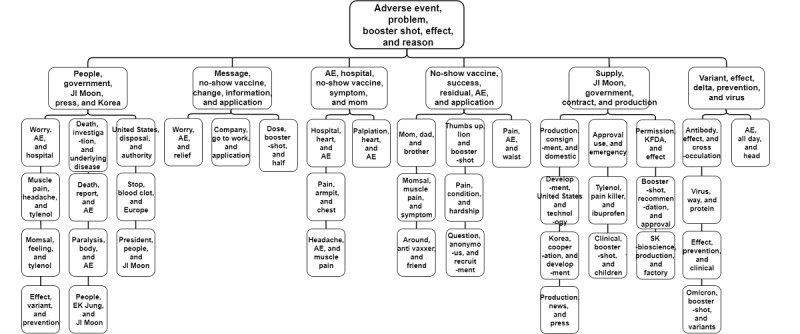
Hierarchical topic modeling of the COVID-19 vaccine discourse on Twitter in Korea. AE: adverse event; KFDA: Korea Food & Drug Administration.

Figure 2 shows the taxonomy of the COVID-19 vaccine discourse organized using hierarchical topic modeling. The root topic words were AE, problem, booster shot, and effect, which were consistent with the major topics in Table 1. Second-level topics were news articles on the Korean government, residual vaccine reservations and vaccination successes, AE, vaccine supply and production, and the effectiveness of vaccines against mutated viruses. Of the 16 third-level topics, 8 (50%) were related to concerns about or experiences with AEs. Other topics included vaccine production, approval, and permission. Another third-level topic was vaccine administration.

### Sentiment Analysis

**Figure 3:**
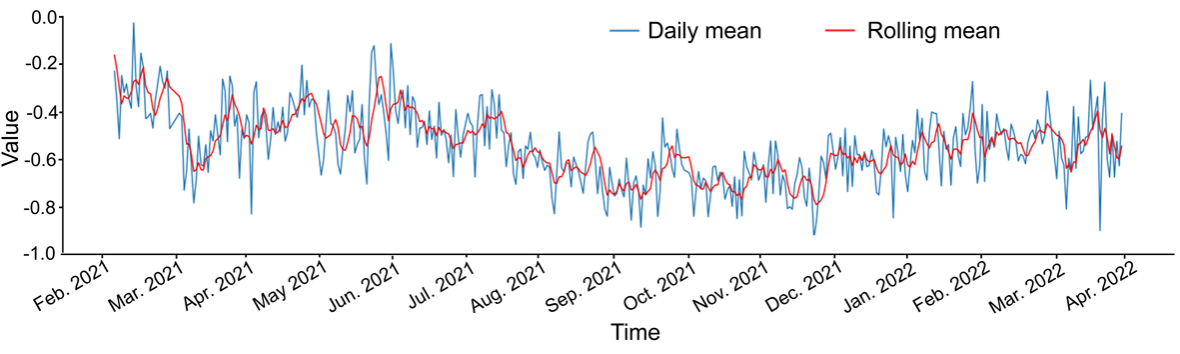
Time trend of sentiment score about COVID-19 vaccine on Twitter in Korea.

Figure 3 show the sentiment analysis results for Twitter posts related to COVID-19 vaccines, in response to research question 2. Overall, negative sentiments regarding COVID-19 prevailed during the study period. The average sentiment score was lowest from July 2021 to December 2021. The Chow test statistics were highly significant for break points in July 2021 and January 2022 when vaccination was intensively administered to the general population.

The research question 3 is about specific topics with respect to vaccine brands.

Approximately 60% (95,857/165,984) of the tweets mentioned ≥2 vaccine brands together. Thus, tweets were classified into topics by vaccine brands based on the highest topic probability calculated by pinning topic modeling to explore public opinion (Table 2). Topics related to each vaccine brand differed. The topic words for the “Pfizer” brand were related to vaccine AEs (eg, muscle pain, headache, menstruation, and pain). The topics regarding the “AstraZeneca” brand involved vaccine effectiveness against virus variants (eg, effectiveness, variant, antibody, booster shot, and prevention) and AEs (eg, AEs and thrombus). The topic words for the “Moderna” and “Janssen” brands were commonly related to vaccine access (eg, no-show vaccine, reservation, and booster shot). The topics related to the “Novavax” brand were vaccine production, supply, and the Korean government. The Korean president’s name, “Jae-in Moon,” and the keyword “president” were the top topics for Novavax. Unspecified topics without weighting the vaccine brand name were keywords related to concerns or reports on AEs, such as death, AE, and health.

The average sentiment score of topics with keywords related to vaccine AEs was significantly lower than that of the other topics (ANOVA and Tukey post hoc test, *P*<.05, data not shown). For example, the sentiment score of the pining topic of the Pfizer brand was >3 times worse than that of Janssen.

**Figure 4:**
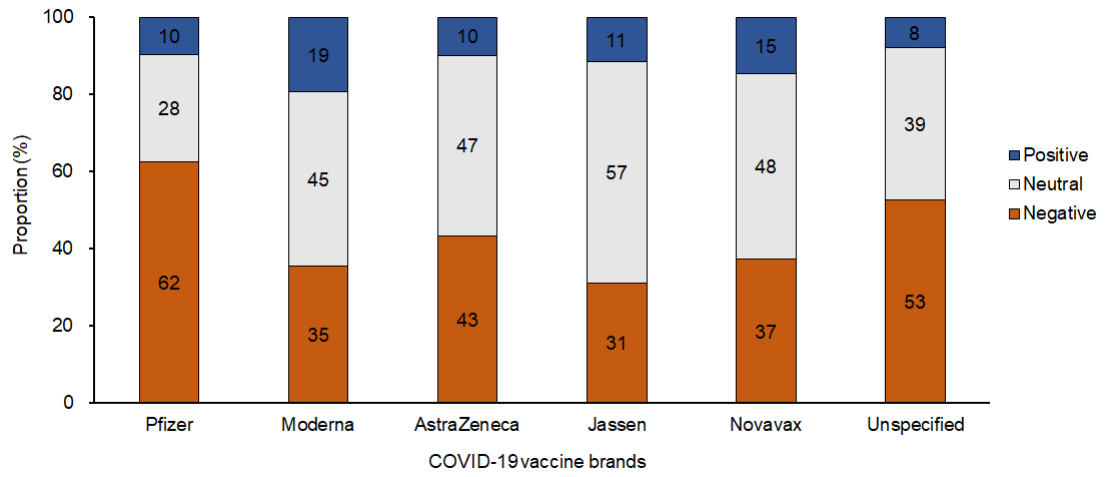
Proportions of positive, neutral, and negative sentiments by COVID-19 vaccine brands.

**Figure 5:**
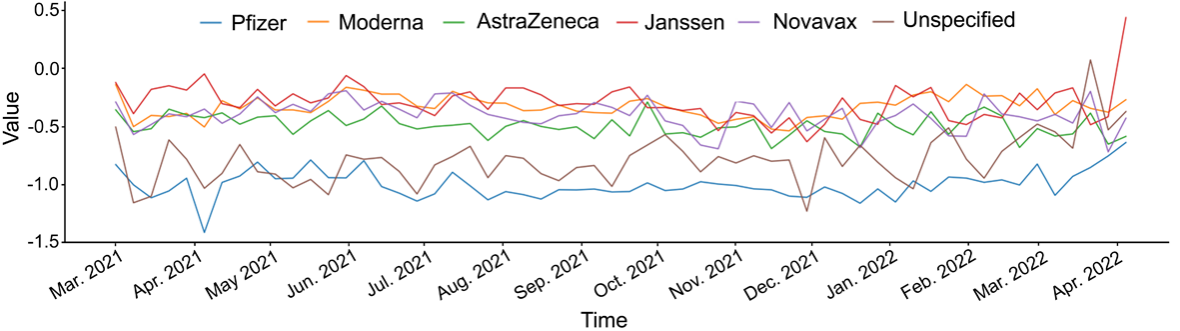
Time trend of sentiment score by COVID-19 vaccine brands.

Figures 4 and 5 show the sentiment distribution for the various vaccine brands. These results are related to research question 4. Similar to the average sentiment score of topics by vaccine brands, the Pfizer brand showed the highest proportion of negative sentiment among the 6 vaccine brands (Figure 4). The average sentiment score with respect to vaccine brand did not show a significant change over time (Multimedia Appendix 6; Figure 5).

**Table 2:** Top 20 topic words and average sentiment scores for various COVID-19 vaccine brands on Twitter in Korea (N=165,984).

Topics	Proportion, n (%)^a^	Topic words	Average sentiment score, mean (SE^b^)
Topic 0: Pfizer	46,274 (27.88)	AE^c^, muscle pain, Tylenol, symptoms, headache, momsal^d^, pain, menstruation, ache, and progress	−1.04 (1.31)
Topic 1: Moderna	53,524 (32.25)	No-show vaccine, AE, booster shot, hospital, mom, worry, friend, dad, cross-vaccination, and doctor	−0.33 (1.20)
Topic 2: AstraZeneca	22,045 (13.28)	Effectiveness, AE, variant, booster shot, prevention, approval, blood clot, antibody, United States, and virus	−0.48 (0.97)
Topic 3: Janssen	13,555 (8.17)	Hospital, no-show vaccine, inoculation, United States, application, booster shot, prevention, confirmation, advance reservation, and civil defense	−0.28 (0.90)
Topic 4: Novavax	23,506 (14.16)	Government, Korea, supply, production, Moon Jae-in, United States, people, contract, Japan, and secure	−0.37 (1.02)
Topic 5: unspecified	7080 (4.27)	Death, AEs, health, report, adverse reaction, women, causality, heart, examination, and investigation	−0.82 (1.17)

^a^This represents the number of tweets assigned to the topic with the highest probability because 1 tweet has >1 topic.

^b^All mean values between COVID-19 vaccine brands exhibit significant differences according to ANOVA and Tukey post hoc test at *P*<.05.

^c^AE: adverse event.

^d^The word “Momsal” is a condition caused by extreme fatigue in which one’s body aches and suffers from exhaustion or fever.

### AE-Centric Topic Modeling

We conducted a subgroup analysis of tweets with terms related to AEs, including side effects, symptoms, AE, and AE reporting (Table 3). This analysis aimed to further understand the AE-related posts on Twitter in Korea (research question 5). The analyzed topics revealed a wide range of AE-related issues posted on Twitter in Korea. Some of the topics were presented as AEs related to the COVID-19 vaccination. Systemic and local AEs were observed, which were already analyzed in the topic modeling using all tweets. In addition, this subgroup analysis revealed heart-related AEs (palpitations and myocarditis or pericarditis), changes in appetite or sleep, and irregular menstruation. In this analysis, not only experienced AEs but also various related discourses were confirmed. For example, the discourse posted on Twitter in Korea about the death of the former president (Doo-hwan Chun), who died of leukemia, was suspicious of the AE of COVID-19 vaccination. Moreover, topics related to information delivery were also observed, such as the preventive effects of booster shots against the Omicron variant and health care workers’ (hospital, physician, and nurse) information and explanation.

**Table 3:** Topics of tweets with terms related to adverse events (AE; N=15,371).

Topics	Proportion, n (%)^a^	Topic words
Topic 0: systemic reaction	3839 (24.98)	Muscle pain, headache, Momsal^b^, pain, aches, mild fever, chills, fever, fatigue, and cold
Topic 1: local allergic reaction	931 (6.06)	Armpit, hive, chest, leg, allergy, lymph node, skin, numbness, rash, and lump
Topic 4: palpitation	612 (3.98)	Heart, exercise, palpitation, man, woman, chest, caffeine, coffee, overwork, and eyesight
Topic 3: myocarditis and pericarditis	1115 (7.25)	Heart, chest, emergency room, myocarditis, pain, dyspnea, chest pain, pain killer, pericarditis, and allergy
Topic #4: irregular menstruation	1121 (7.29)	Irregular bleeding, menstrual pain, vaginal bleeding, menstrual cycle, menstrual irregularity, anxiety, premenstrual syndrome, bleeding, due date, and menstrual volume
Topic 5: changes in appetite and sleep	1522 (9.9)	Appetite, fatigue, explosion, insomnia, increase, sleep, hunger, sleepiness, digestion, and improve
Topic 6: suspicion of serious side effects	383 (2.49)	Death, suspicion, blood cancer, Doo-hwan Chun^c^, government, leukemia, AE, dad, health, and cerebral hemorrhage
Topic 7: case report about death or thrombosis	1412 (9.19)	Death, myocarditis, occurrence, case, report, blood clot, approval, thrombosis, risk, and death
Topic 8: discontinuation of vaccination	456 (2.97)	United States, Korea, government, problem, article, disposal, health authority, Japan, suspension, and order
Topic 9: effectiveness of vaccines	381 (2.48)	Effect, antibody, booster shot, infection, immunity, variant, confirmation, prevention, virus, and Omicron
Topic 10: information on side effects	760 (4.94)	Hospital, AE, symptom, physician, no-show vaccine, phone, talk, information, explanation, and nurse
Topic 11: concern of vaccination	2839 (18.47)	Worry, mom, booster shot, friend, relief, suffering, dad, cross-vaccination, brother, and family

^a^This represents the number of tweets assigned to the topic with the highest probability because 1 tweet has >1 one topic.

^b^The word “Momsal” is a condition caused by extreme fatigue in which one’s body aches and suffers from exhaustion or fever.

^c^The word “Doo-hwan Chun” is the name of the former president.

## Discussion

### Principal Findings

This study showed that a wide range of topics regarding COVID-19 vaccines have been discussed on Twitter in Korea. The topics related to the COVID-19 vaccine were vaccine-related AEs; emotional reactions such as worries and appreciation for vaccination; vaccine development, supply, or application; and government vaccination policies. Among them, the most important and frequently mentioned topic was AEs related to COVID-19 vaccination. Vaccine-related AEs included systemic and local AEs, myocarditis or pericarditis, thrombus, irregular menstruation, changes in appetite and sleep, leukemia, and death. A topic modeling study in which weights were assigned to various vaccine brands found notable differences in the topics related to the various vaccine brands. The topics pertaining to the Pfizer vaccine mainly mentioned AEs, those related to Moderna and Janssen vaccines focused on vaccine access, those pertaining to AstraZeneca were related to vaccine effectiveness, and those regarding Novavax were issues related to vaccine production and supply. Although the sentiments toward COVID-19 vaccines changed over time, negative sentiments prevailed since the start of the vaccination. In terms of vaccine brands, the topics pertaining to the Pfizer vaccine expressed the strongest negative opinion.

### Comparison With Prior Work

The diffusion of new technologies changes the methods for data collection or the analysis of people’s thoughts, feelings, and actions [42]. Opinions expressed on social media provide researchers with alternative sources of qualitative and quantitative information to complement or, in some cases, provide alternatives to traditional data collection methods [43]. From this perspective, the scientific method of information epidemiology has gained increasing attention for assessing public health perceptions and health status using web-based data sources [44]. Recently, there has been an increasing number of studies on social media posts, and the most popular platform among them is Twitter [44,45].

In this context, research on public discourse and opinions on COVID-19 vaccines has mainly used Twitter data [4,5,11,12,18,23,27]. Therefore, we investigated topics and sentiments related to COVID-19 vaccines using tweets. The overall topic composition was similar to that of previous studies, which included vaccine safety and efficacy, vaccine development and supply, and national vaccination policies. However, a subgroup analysis focusing on AEs allowed for a more in-depth analysis of topics related to various side effects. The results of this study showed that some topics about AEs were consistent with side effect profiles obtained from phase 3 clinical trials previously reported for messenger RNA (Pfizer and Moderna) [15,46] and adenovirus vector vaccines (Jassen and AstraZeneca) [14,47]. Systemic reactions (eg, myalgia, headache, fatigue, pyrexia, and chills) and local reactions (eg, injection site pain) were common AEs after COVID-19 vaccination.

This study identified topics for other side effects that were not reported in the preapproval clinical trials. For example, changes in menstruation, appetite, and sleep have been reported. Reports on sleep changes are rare. One study showed that sleep duration increased after vaccination based on wearable device data [48]. Changes in the menstrual cycle and unexpected vaginal bleeding are not listed in the safety profile, but there have been reports of people experiencing these symptoms immediately after vaccination [49]. Although the evidence is limited, recent studies have determined the association between menstrual changes and COVID-19 vaccination [50]. Public concern is increasing because irregular menstruation can affect fertility [51]. This study revealed the experiences of menstrual cycle changes in Korean women. Further studies are required to determine the causal relationship between menstrual cycle changes and COVID-19 vaccination.

In addition, Korean tweets indicated public suspicion of vaccination and the occurrence of leukemia. To date, there has been no evidence of an association between leukemia and COVID-19 vaccination. However, many Korean tweets mentioned that the occurrence of leukemia in the former President Doo-hwan Chun could be related to COVID-19 vaccination. Vaccine-related misinformation on social media platforms may exacerbate vaccine hesitancy [52]. To prevent the spread of misinformation, the government needs to understand the public opinions expressed on social media and respond appropriately.

Several other studies that analyzed sentiments toward COVID-19 vaccines reported positive public opinion [4,10,11,13,23,25-27]. However, this study showed that public opinion was consistently negative toward COVID-19 vaccination over 1 year since the start of vaccination. Negative sentiments were most pronounced when vaccination was initiated in people in their 20s and 50s. Moreover, negative public opinion was maintained on Twitter in Korea until >80% (approximately 41 million/51 million) of the population was vaccinated. Our results agree with the limited number of previous Korean studies conducted during the early stages of vaccination. In a survey conducted at the start of vaccination, more than half of the population hesitated to receive the vaccine [53]. A study analyzing Twitter data during the first month of vaccination showed that public sentiment toward the vaccine was negative in Korea [19].

Two possible explanations for these results are the compulsory vaccination policy and the experiences and concerns regarding AEs after vaccination. After the Omicron variant epidemic, the Korean government permitted only fully vaccinated people to use public places, such as restaurants, cafes, and movie theaters. Similarly, many nations have adopted mandatory vaccinations, including Australia, Brazil, Canada, France, Indonesia, Italy, and the United Kingdom [54]. In France, approximately half of the respondents opposed such a policy [55]. Thus, a compulsory vaccination policy might worsen public opinion in Korea. Another reason is that concerns or experiences of adverse effects after vaccination may exacerbate negative sentiments. In addition, suspicions about death and serious AEs (eg, leukemia) might have affected these sentiments. The results of this study showed that topics with many keywords related to AEs had lower sentiment scores than others. Considering the continuous evolution of variant viruses and the short duration of immunity of vaccines, additional vaccination may be necessary [1,2]. The Korean government should manage misinformation and provide accurate information related to AEs to decrease the negative public opinion.

The sentiment analysis by vaccine brands suggests that the Pfizer brand had the strongest negative score among the 5 vaccine brands, which was inconsistent with the previous results of phase 3 clinical trials and postmarketing surveillance. Initial trials of Pfizer revealed no significant differences in side effects compared with other vaccine brands. Rather, it was more effective in preventing symptomatic COVID-19 than the AstraZeneca and Jassen vaccines [14,15,46,47]. The postmarketing surveillance showed that severe AEs, such as deaths, for Pfizer and Moderna were similar [56]. This discrepancy between academic evidence and public opinion may be because Pfizer vaccine was first inoculated in older people and patients with underlying disease in Korea. Therefore, the Pfizer vaccine may have more reported side effects in the early period of vaccination, which could affect the negative sentiment. Furthermore, cross-vaccination mainly used the Pfizer vaccine as the second dose. Although there have been controversial results in terms of the association between cross-vaccination and the severity of side effects [57], people are more likely to be concerned about cross-vaccination compared with the same vaccine inoculation. Thus, the circumstances regarding Pfizer vaccination in Korea may affect negative sentiments.

To the best of our knowledge, few studies have comprehensively analyzed Korean tweets more than a year after the start of vaccination to determine people’s opinions and perceptions of COVID-19 vaccines. Our topic analysis provides a hierarchical view of the topics related to COVID-19 vaccines that are mainly discussed on a web-based social media platform. Moreover, we tracked the trend of sentiments toward COVID-19 vaccines over time. We also conducted quarter-based topic analyses to reflect the rapidly changing COVID-19 circumstances. Finally, we carefully refined the Korean tweets using various preprocessing methods to obtain high-quality results.

### Limitations

First, caution is needed in interpretation because the relationship between vaccination and response was not analyzed and the topics about AEs did not represent a causal relationship. Second, this study only used the Twitter data. Thus, other social media platforms may contain different opinions because their preferences may vary depending on user characteristics. Third, because most social media users were young adults, our findings may not reflect the views of the entire population. Fourth, our sentiment analysis relied on English translation because of the absence of an adequate tool suitable for the Korean language. Thus, sentiment scores may have been significantly influenced by the success of translation. Furthermore, we dealt only with nouns for topic modeling. Other parts of speech, such as adjectives, adverbs, and verbs, will be considered in future studies. Finally, our sentiment analysis was performed after English translation because of the dependency on the SentiStrength program. Although we double-checked that the translation did not affect the quality of the sentiment analysis results, there could be ambiguity and uncertainty in the translation process, as indicated in the study by Huang et al [58].

### Conclusions

Our results showed persistent public discourses about AEs after vaccination and predominantly negative sentiments on Twitter in Korea. These results suggest that accurate information regarding vaccine-related AEs should be communicated to the general public. In addition, a continuous analysis of public opinion, not a one-time event, is required, and crisis communication should be continuously conducted according to public opinion changes. In particular, the Pfizer vaccine had the most negative sentiment from the early period of vaccination among the five vaccine brands, showing that public opinion is not based on academic evidence. Misinformation on web-based platforms should be controlled properly from a public health perspective. Furthermore, this study on public discourse and opinions after large-scale vaccination over a short period can be a valuable resource for responding to outbreaks of other emerging infectious diseases.

## Appendix

Multimedia Appendix 1 Summary of topics and sentiments from previous studies on COVID-19 vaccines using social media data.

Multimedia Appendix 2 Detailed algorithms and descriptions of each phase of COVID-19 vaccine discourse analysis.

Multimedia Appendix 3 A list of excluded Twitter accounts.

Multimedia Appendix 4 The methods for selecting the optimal number of topics in latent Dirichlet allocation.

Multimedia Appendix 5 Comparison of sentiment analysis using original Korean texts and translated English texts.

Multimedia Appendix 6 The results of autoregression between sentiment score and time by Covid-19 vaccine brands.

## Abbreviations

AE: adverse event
LDA: latent Dirichlet allocation
LI: label intrusion
OL: optimal label
TF-IDF: term frequency–inverse document frequency
