# Supplementary material for: A Comprehensive Analysis of COVID-19 Vaccine Discourse by Vaccine Brand on Twitter in Korea: Topic and Sentiment Analysis
Source: J Med Internet Res. 2023 Jan 31;25:e42623. doi: 10.2196/42623 (PMC9891356; doi:10.2196/42623)
Supplement: Multimedia Appendix 1 [file jmir_v25i1e42623_app1.docx]

 Multimedia Appendix 1. Summary of topics and sentiments from previous studies on COVID-19 vaccines using social media data.

| **Author** | **Data source** | **Region** | **Method** | **Main topics** | **Sentiments** | **Time period** |
| --- | --- | --- | --- | --- | --- | --- |
|  |  |  |  |  |  |  |
| Huangfu 2022 [4] | Twitter | Not restricted by region; language was English. | Topic modeling (LDA), sentiment analysis (VADER) | Each topic was analyzed according to the polarity of emotions. Topics of positive emotions were: planning for getting vaccination (62.1%) and getting vaccination (18.8%). Topics of negative sentiments were: vaccine hesitancy (56.0%), extreme side effects (9.6%), and vaccine supply and rollout (8.3%). | Positive 46.5%, negative 23.8%, neutral 28.7%. Negative sentiment trends were stable; positive sentiments could be easily influenced. | 12/14/2020–4/30/2021 |
| Karami 2021 [5] | Twitter | USA | Topic modeling (LDA), sentiment analysis (LIWC, VADER) | The vaccine sites and the vaccination and election were the most and least popular topics, respectively. There was high discussion on topics related to vaccination hesitancy and immunity, indicating these are priority topics for Twitter users. | Negative: 33.64%, non-negative: 66.36%. The rate of negative tweets had a decreasing trend and the rate of non-negative tweets had an increasing trend during the period. | 11/01/2020–2/28/2021 |
| Liew 2021 [6] | Twitter | Not restricted by region; language was English. | Topic modeling (STM), sentiment analysis (VADER) | Major topics were emotional reactions related to COVID-19 vaccines (19.3%), public concerns related to COVID-19 vaccines (19.6%), discussions about news items related to COVID-19 vaccines (13.3%), public health communications about COVID-19 vaccines (10.3%), discussions about approaches to COVID-19 vaccination drives (17.1%), and discussions about the distribution of COVID-19 vaccines (20.3%). | Negative sentiments, emotional reactions, public concerns related to COVID-19 vaccines. | 11/18/2020–2/3/2021 |
| Monselise 2021 [7] | Twitter | Not restricted by region; language was English. | Topic modeling(LDA, NMF), sentiment analysis (VADER) | Administration (8.7%) and access to vaccines (8.3%) were some of the major concerns. | Positive 33.2% (2,645,705/7,948,886): (joy 21.9%, hopefulness 11.2%); neutral 19.9% (1,582,221/7,948,886); negative 46.9% (3,720,960/7,948,886): (fear 37.8%, sadness 5.1%, anger 3.9%) | 12/16/2020–2/13/2021 |
| Zhang 2021 [8] | Twitter | USA | Topic modelling (LDA), sentiment analysis, linear regression analyses, network analysis and visualization | Among 12 topics, the topic on vaccine development and people’s views (like count β: .055, retweet count β: .078) and vaccine efficacy and rollout (like count β: .049, retweet count β: .077) had relatively larger effects.   The 2500 most liked and most retweeted retweets clustered around the topics on vaccine access, vaccine efficacy and rollout, vaccine development and people’s views, and vaccination status. | The overall valence of the tweets was positive, with a score of 0.145 | 1/1/2020–4/30/2021 |
| Melton 2021 [10] | Reddit | N/A | Topic modeling(LDA), sentiment analysis | Topic modeling revealed community members mainly focused on side effects rather than outlandish conspiracy theories. Topics 1–4 appeared to be closely related to a broader discussion on vaccine, safety concerns, efﬁcacy, and potential side effects. Topic 5 appeared to be focused on much broader terms and information (i.e., news, sources, questions) and direct mention of concerns about vaccination. | Positive (56.68%), negative (27.69%), neutral (15.63%). *Polarity analysis suggested these communities expressed more positive sentiment than negative regarding vaccine-related discussions and has remained static over time. | 12/1/2020–5/15/2021 |
| Hu 2021 [11] | Twitter | USA | Topic modeling (LDA), sentiment analysis (VADER, NRCLex) | COVID-19 testing, waiting for vaccine, Bill Gates, vaccine development, effects of COVID-19, school reopening, pandemic control, Pfizer, vaccine concerns, pandemic control, Kamala Harris, racism, effectiveness of Pfizer vaccine, vaccine management, presidential election, effectiveness of vaccine, first COVID-19 vaccine, vaccine distribution, concerns about vaccine, vaccination, vaccine appointment, vaccine for detainees, vaccine administration, prioritize teachers for vaccine. | * The emotion with the highest weekly average scores along the majority of the timeline was trust followed by fear, anticipation, sadness, anger, joy, disgust, and surprise. * An increasing trend in positive sentiments in conjunction with a decreasing trend in negative sentiments were generally observed in most states, reflecting the rising confidence and anticipation of the public towards vaccines. | 3/1/2020–2/28/2021 |
| Guntuku 2021 [12] | Twitter | USA | Topic modeling (LDA), logistic regression | December: allergic and adverse reactions, misinformation regarding Bill Gates and China, issues of trust among Black Americans in the healthcare system. January: questions about mask wearing, reaching herd immunity and natural infection, concerns about nursing home residents and workers. February: access to black communities, waiting for appointments, keeping family safe by vacci- nating, and ﬁghting online misinformation campaigns. | N/A | 12/1/2020–2/28/2021 |
| Chandrasekaran 2022 [13] | Twitter | Not restricted by region; language was English | CoreX topic modeling, VADER sentiment analysis, qualitative content analysis | The most tweeted topic about COVID-19 vaccination was related to vaccination policy, specifically whether vaccines needed to be mandated or optional (13.94%), followed by vaccine hesitancy (12.63%), and post-vaccination symptoms and effects (10.44%). | Average compound sentiment scores were positive throughout the 16- week study period. | 1/1/2020–4/30/2021 |
| Boucher 2021 [17] | Twitter | Not restricted by region;  language: English and French. | Social network analysis, topical analysis of cluster | Main themes about vaccine hesitancy conversation were safety, efficacy, freedom, and mistrust in institutions. COVID-19 vaccine hesitancy clusters showed the topics about freedom or mistrust of institutions (45,628/146,191; 31.2%), and criticism toward the government’s handling of the pandemic (34,756/146,191; 23.8%) | N/A | 11/19/2020–11/26/2020 |
| LC Jiang 2021 [18] | Twitter | USA | Topic modeling(LDA) | Seven themes were presented as major topics: news related to coronavirus and vaccine development, general discussion, financial concerns, venting negative emotion, prayers and calls for positivity, efficacy of vaccine and treatment  conspiracies about coronavirus and its vaccines. | N/A | 2/21/2020–3/20/2020 |
| Shim 2021 [19] | Twitter | South Korea | Topic modeling(LDA), sentiment analysis (KNUsenti lexicon) | The major topics were vaccine hesitancy (14.2%), vaccine development (13.1%), quarantine prevention policy (13.0%), vaccine efﬁcacy (12.6%), priority vaccination of hospital workers (12.0%), media on COVID-19 vaccines (11.9%), medical association’s responses (11.8%), and adverse reactions (11.4%). | The sentiment analysis revealed a similar ratio of positive and negative tweets immediately before and after the commencement of vaccinations, but negative tweets were dominated after the increase in the number of confirmed COVID-19 cases. | 2/23/2021–3/22/2021 |
| Luo 2021 [20] | Twitter & Weibo | USA (Twitter), China (Weibo) | Sementic network analysis | Twitter: vaccine promotion and anti-vaccine discourses (39.0%), personal vaccination experience (17.0%). Weibo: vaccination policies and priority groups (40.0%), domestic vaccines research and development (22.0%). | Twitter: neutral (49.99%), positive (30.62%), negative  (19.40%).  Weibo: positive (40.64%), neutral (37.44%), negative (21.92%). | 12/1/2020–2/20/2021 |
| X Jiang 2021 [21] | Twitter | Not restricted by region; language was English | Structural topic modeling (STM), sentiment analysis (BERT) | Liberal Twitter users: vaccine development (27.08%), conspiracy regarding big pharma and inequality (19.46%). Conservative users: conspiracy regarding digital surveillance, mandatory vaccination and other doubts (24.68%), countries and individuals' roles in vaccine development | Twitter users tended to express less favorable vaccine-related sentiments. | 3/1/2020–6/30/2020 |
| Roy 2021 [22] | Twitter | Not restricted by region; language was English. | Machine learning models for sentiment analysis (K-fold cross validation, CNN, naïve Bayes, multiple regression, curve ﬁtting) | Vaccine, mask, social distancing | There was a near-perfect balance between positive and negative sentiments globally. | 05/2020–12/2020 |
| Kwok 2021 [23] | Twitter | Not restricted by region; language was English. | Topic modeling(LDA), sentiment analysis (NRCLex) | Topic 1 was attitudes toward COVID-19 and the vaccines. Topic 2 was advocating infection control measures against COVID-19. Topic 3 was misconceptions and complaints about COVID-19 control measures. | Positive (67%), negative (30%), neutral (3%). | 1/22/2020–10/20/2020 |
| Yan 2021 [24] | Reddit | Canada | Topic modeling (LDA), sentiment analysis | Of the 13 topics, two were related to vaccines: vaccine uptake, vaccine supply.  (1) advocating for restrictions, (2) COVID-19 transmission, (3) impacts of COVID-19 on social spheres, (4) discussion about case numbers, (5) outbreaks in health care facilities, (6) debating how realistic public health orders are, (7) scientific concepts surrounding COVID-19, (8) monitoring travelers and people who have been exposed, (9) violating and enforcing restrictions, (10) vaccine uptake, (11) general speculations, (12) impact on hospitals, and (13) vaccine scarcity. | Vaccine-related comment: Vancouver 1) From July 2020 to April 2021: Negative (anger) > joy 2) From April 2021 onward: mean scores for joy began to increase, mean scores for negative emotions decreased.  Sentiment scores for vaccine uptake differed across the 3 cities (P < .001). | 7/13/2020–6/14/2021 |
| Lyu 2021 [25] | Twitter | Not restricted by region; language was English. | Topic modeling(LDA), sentiment and emotion analyses (NRCLex) | Opinions about vaccination (227,840 tweets, 15.2%) waere the most tweeted topic. Vaccine development worldwide (83,156 tweets, 5.55%) became the most discussed topic around August 11, 2020. The topic on instructions on getting vaccines (106,544 tweets, 7.11%) gradually became more salient and most discussed topic after the first week of January 2021. | Sentiment was increasingly positive in general. Trust was the most predominant emotion, followed by anticipation, fear, and sadness. | 3/11/2020–1/31/2021 |
| Liu 2021a [26] | Twitter | Not restricted by region; language was English. | Sentiment analysis (VADER): temporal, geographic |  | Positive: 42.8%, neutral: 26.9%, negative: 30.3% | 11/1/2020–1/31/2021 |
| Hussain 2021 [27] | Facebook, Twitter | UK, USA | Sentiment analysis (VADER, TextBlob, BERT) | N/A | United Kingdom: positive: 58%, negative: 22%, neutral: 17%. USA: positive: 56%, negative: 24%, neutral: 18%. | 3/1/2020–11/22/2020 |
| Liu 2021b [28] | Twitter | USA | Topic modeling(LDA) | This study did not interpret specific topic contents. They just showed the stance of topics (e.g., positive and negative attitudes and behavioral intentions). | N/A | 11/1/2020–1/31/2021 |
| Cotfas 2021 [29] | Twitter | UK | Sentiment analysis (annotation, RF/SVM/LSTM/BERT) | N/A | Against (negative): 14.38%, neutral: 68.90%, in favor (positive): 16.72% | 11/9/2020–12/8/2020 |
| Marcec 2022 [30] | Twitter | Not restricted by region; language was English | Sentiment analysis (AFINN lexicon): vaccine type | N/A | Pfizer and Moderna vaccines: positive and stable; AstraZeneca/Oxford vaccine: decreasing, positive (December), negative (March). | 12/1/2020–3/31/2021 |
