# Supplementary material for: A Comprehensive Analysis of COVID-19 Vaccine Discourse by Vaccine Brand on Twitter in Korea: Topic and Sentiment Analysis
Source: J Med Internet Res. 2023 Jan 31;25:e42623. doi: 10.2196/42623 (PMC9891356; doi:10.2196/42623)
Supplement: Multimedia Appendix 2 [file jmir_v25i1e42623_app2.docx]

**Multimedia Appendix** **2. Detailed algorithms and descriptions of each phase in Figure 1**

In this appendix, we provide detailed algorithms and descriptions of each component in Figure 1.

### Data Collection

Textbox 1 presents an algorithm for Twitter data collection. In this algorithm, we used three input parameters: the specified start date (*S*), the specified end date (*E*), and a list of Korean keywords (*K*) (Line 1). Using these parameters, we established a set of query parameters (*Q*) (Line 2). *Q* includes the specified period from *S* to *E*, Twitter language (i.e., lang), tweet creation date (i.e., created_at), keyword list (*K*), tweet author identifier (i.e., author_id), and geolocations (i.e., geo.place_id and country). Using *Q*, we made an API call to Twitter to collect Korean tweets written in the specified data range (from *S* to *E*). We then received an initial dataset (*D_init_*) in response to the API call (Line 3). We could not use *D_init_* because it contained unnecessary emoticons that caused encoding errors. Thus, we refined the dataset (*D*) by removing emoticons from *D_init_* (Line 4). Then, we reformatted *D* in the JSON format and returned *J* for further processing (Line 5).

Textbox 1. Data collection algorithm (*TwitterCrawling*) using the Twitter API.

| **Input**: Start date *S*, End date *E*, list of Korean keywords *K*  **Output**: JSON file, *J*  Line 1: **Function**: *TwitterCrawling*(*S*, *E*, *K*)  Line 2: *Q* = *initQueryParam*(*S*, *E*, *K*);  Line 3: *D_init_* = *requestAPI*(*Q*);  Line 4: *D* = *removeEmoticons*(*D_init_*);  Line 5: *J* = *buildJSON*(*D*);  Line 6: **return** *J*;  Line 7: **End Function** |
| --- |

### Data Preprocessing

Textbox 2 shows the preprocessing algorithm for the Korean Twitter dataset. The function (termed *Preprocessing*) for implementing the algorithm used the input JSON file (*J*) obtained from the algorithm described in Textbox 1 and a list of given keywords (*K*) (Line 1). We first created a data frame (*C*) with columns to store the tweet creation timestamp and preprocessed tweets (Line 2). We then extracted the tweets from the given J (Line 3). For each tweet ($T_{i}$) in *T*, we applied a filter, through which a potentially valid tweet was left and collected into C (Lines 4–5). The filter was used to perform five inspections. First, if a given tweet was a retweet, the tweet was eliminated. Second, it removed any tweets, such as an official tweet written by a government institution account and a tweet uploaded by a disaster alert bot that is not authored by the general public. Third, it eliminated any tweets related to news articles or advertisements. Fourth, it eliminated ad tweets that included any keyword in the given list (*K*). Fifth, it dropped tweets containing keywords with different meanings from the given keywords. We then removed any web link and replaced various synonyms with common representative words in the retained tweets (Lines 6–9). As a sanity check, if the retained tweet was an empty string or contained fewer than two words, we discarded that tweet (Line 10. Otherwise, we collected the surviving tweet in C for further processing (Lines 11–12). The running algorithm removed duplicates in C and returned the retained tweets (Lines 13–15). Finally, we retained and used a total of 165,984 tweets for this study.

Textbox 2. Data preprocessing algorithm. The approach applied various filters to remove invalid tweets.

| **Input**: A JSON file *J* that has a set of matching tweets and list of keywords (K)  **Output**: A preprocessed file, *F*  Line 1: **Function**: *Preprocessing*(*J, K*)  Line 2: *C* <- Initialize a data frame.  Line 3: *T* <- Extract tweets from the given JSON file, *J*.  Line 4: **For** $T_{i} \in T$  Line 5: **IF** (*isTweetValid*(${K, T}_{i}$) == false) **Then** **continue**;  Line 6: **Else**  Line 7: Remove a hyperlink in $T_{i}$.  Line 8: Replace synonyms with one representative word in $T_{i}$.  Line 9: **End Else**  Line 10: **IF** ${(T}_{i}$ is an empty string or contains fewer two words) **Then continue;**  Line 11: **Else** C.add($T_{i}$)  Line 12: **End For**  Line 13: *F* <- Remove any duplicates in *C*.  Line 14: **return** *F*;  Line 15: **End Function** |
| --- |

### Topic Modeling Analysis: Latent Dirichlet Allocation (LDA)

Textbox 3 describes the LDA algorithm that was applied to the dataset. The algorithms take a set (*D*) of documents preprocessed in Textbox 1 and the number (*k*) of topics (Line 1). We then initialized a data frame for an LDA input corpus (Line 2). For each preprocessed document ($D_{i}$), the algorithm performs a morpheme analysis and extracts parts-of-speech tagged as ‘NNG’ and ‘NNP’ from that analysis (Lines 4–5). We then removed the specified vaccine brand names (Pfizer, Moderna, AstraZeneca, Janssen, and Novavax) to avoid affecting our analysis (Line 6). We built an LDA model with the constructed corpus (C) and the specified number of top topics (*k*) and trained the model 1,000 times (Lines 9–10).

Textbox 3. LDA algorithm. We trained the LDA model 1000 times.

| **Input**: A set of preprocessed documents *D* and the number of topics *k*  **Output**: LDA model, *M*  Line 1: **Function**: *LDA(D*, *k*)  Line 2: *C* <- Initialize a data frame.  Line 3: **For** $D_{i} \in D$  Line 4: *M*$A_{i}$ = morphAnalysis($D_{i})$  Line 5: $N_{i}$ = extractNouns(*M*$A_{i}$*)*  Line 6: $N_{i}$ = removeVaccineBrands($N_{i})$  Line 7:  *C.*add($N_{i}$)  Line 8: **End For**  Line 9: *M* = LDA(*C,k)*  Line 10: *M.*train(iterations=1000)  Line 11: **return M**  Line 12: **End Function** |
| --- |

### Hierarchical Topic Modeling Analysis: HLDA

Textbox 4 illustrates the hierarchical topic modeling analysis (HLDA) function. We considered two parameters: a set of processed documents (D) and a given depth (*d*) (Line 1). Lines 2–8 are the same as those in Textbox 3. In Lines 9–10, we ran the HLDA with the constructed corpus (C) and *d* and trained the model 1000 times. By using freeze_topics, we prevented the number of topics from rapidly increasing by restricting new topic creation during training.

In Line 11, we applied topic pruning to the trained model because too many topics can make our interpretation more difficult. For this pruning, we selected the top *K* topics at depth 1, based on the number of documents associated with each topic. For each topic, we selected the top three subtopics. That is, we checked if the number of documents belonging to a subtopic was greater than a threshold (i.e., 0.2% of the total documents in our dataset), and that subtopic became a candidate for that selection. We sorted the candidates by the order of their document counts and then selected the top three.

Finally, we returned the pruned and refined HLDA model (Lines 12–13).

Textbox 4. HLDA algorithm. We applied pruning for better interpretation.

| **Input**: A set of preprocessed documents *D*, the depth *d*, and the number of topics *K*  **Output**: The HLDA model *M*  Line 1: **Function**: *getHLDAModel*(*D*, *d*)  Line 2: *C* <- Initialize a data frame.  Line 3: **For** $D_{i} \in D$  Line 4: *M*$A_{i}$ = morphAnalysis($D_{i})$  Line 5: $N_{i}$ = extractNouns(*M*$A_{i}$*)*  Line 6: $N_{i}$ = removeVaccineBrands($N_{i})$  Line 7:  *C.*add($N_{i}$)  Line 8: **End For**  Line 9: *M* = HLDA(*C, d)*  Line 10: *M.*train(iterations=1000, freeze_topics = True)  Line 11: *M* <- applyingPrunning(*M*, *K*)  Line 12: **return** *M*  Line 13: **End Function** |
| --- |

Textbox 5 describes the function (*sentimentAnalysis*) for our sentiment analysis using a Python package called sentistrength (v0.0.9). To use this package, we configured the paths for a necessary jar file (SentiStrengthCom.jar) and a language folder. This function takes a set of preprocessed documents (or tweets) (*D*) (Line 1). We then initialized a list (*S*) to contain the sentiment scores (Line 2). Each preprocessed document ($D_{i}$) was translated into English for subsequent analysis (Lines 3–4). We obtained the positive and negative sentiment scores scores for the translated document via getSentiment() in sentistrength (Line 5). We summed both scores and added the results to *S* (Line 6). Once the sentiment scores of all documents were computed, S was finally returned.

Textbox 5. Sentiment analysis. We utilized the sum of the negative and positive scores for each document.

| **Input**: A set of preprocessed documents *D*  **Output**: A list of sentiment scores *S*  Line 1: **Function**: *sentimentAnalysis*(*D*)  Line 2: *S* <- Initialize a list to store sentiment scores.  Line 3: **For** $D_{i} \in D$  Line 4: $D_{i}'$<- Translate $D_{i}$ to English.  Line 5: $S_{i}$ <- getSentiment($D_{i}'$, score=‘dual’)  Line 6: *S.*add($S_{i}$[0] + $S_{i}$[1])  Line 7: **End For**  Line 8: **return** *S*  Line 9: **End Function** |
| --- |
