# Supplementary material for: A Comprehensive Analysis of COVID-19 Vaccine Discourse by Vaccine Brand on Twitter in Korea: Topic and Sentiment Analysis
Source: J Med Internet Res. 2023 Jan 31;25:e42623. doi: 10.2196/42623 (PMC9891356; doi:10.2196/42623)
Supplement: Multimedia Appendix 4 [file jmir_v25i1e42623_app4.docx]

**Multimedia Appendix 4. The methods for selecting the optimal number of topics in LDA**

In this appendix, we provide detailed methods for selecting the optimal number of topics in LDA

Given that (i) the spike appears around 50 on the x-axis in the coherence plot (left in Figure 1) and (ii) a sharp rise emerges from 50 to 60 in the perplexity plot (right in Figure 1), 50 seems as the most appropriate number of topics in our study.


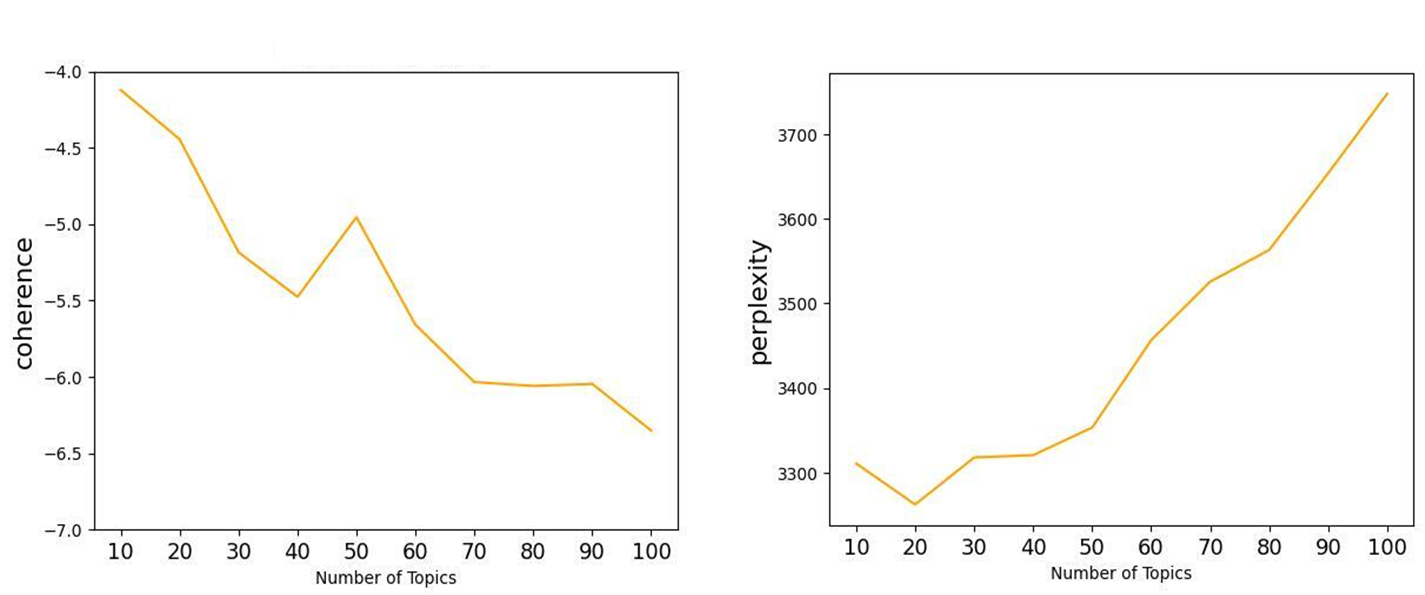


**Figure 1. Coherence and perplexity values over the increasing number of topics.**

Furthermore, we calculated some other indicators for the best number of topics in our dataset as shown in Figure 2. From this plot we could observe that the optimal number of topics was in the range 40 – 60. Hence, we reached a conclusion that it was a reasonable choice to select 50 as the optimal number of topics for our dataset. Seeking for a better number is left as future work.


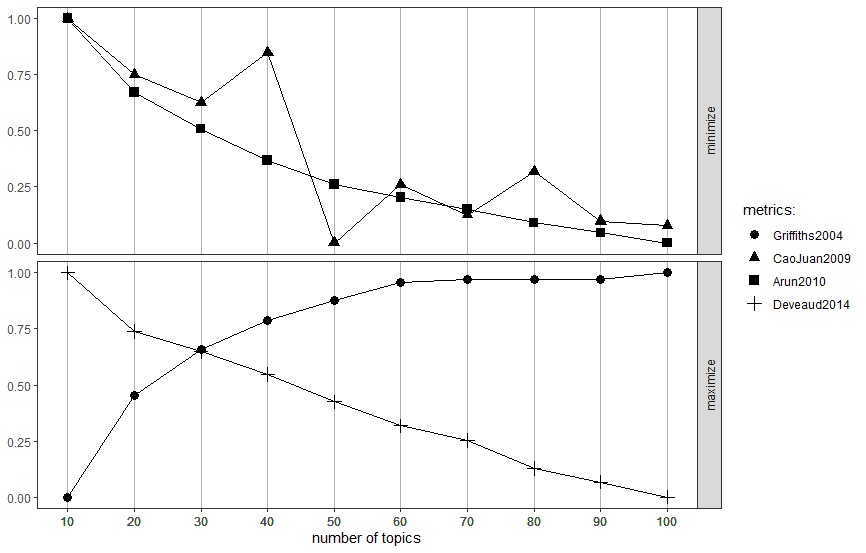


**Figure 2. Results from the metrics of Arun2010, CaoJuan2009, Deveaud2014, and Griffiths2004. The best number of topics seems in the range 40-60.**
