# Supplementary material for: A Comprehensive Analysis of COVID-19 Vaccine Discourse by Vaccine Brand on Twitter in Korea: Topic and Sentiment Analysis
Source: J Med Internet Res. 2023 Jan 31;25:e42623. doi: 10.2196/42623 (PMC9891356; doi:10.2196/42623)
Supplement: Multimedia Appendix 5 [file jmir_v25i1e42623_app5.docx]

 Multimedia Appendix 5. Comparison of sentiment analysis using original Korean texts and translated English texts.

In this appendix, we provide our process of comparing results of sentiment analysis using original Korean texts and translated English texts.

SentiStrength also supports sentiment analysis for Korean text. However, one challenge we encountered when using the SentiStrength program was that the program's sentiment analysis results on most of the Korean tweets in our dataset were neutral (147820, approximately 90% of total tweets), which was incorrect when we compared the results to what we analyzed. We used the original tweet's translation into English to address this issue. As a result, we found that the program's analysis quality had significantly improved.

Table 1 compares the sentiment analysis results between the original and the corresponding translated tweet. The original Korean tweet contains primarily negative and a few positive comments about Moderna's effect after the second dose; the result was close to neural. On the contrary, the SentiStrength program produces reasonably accurate positive and negative scores for the translated text. As a result, we discovered that our decision to use the translation for SentiStrength was correct.

Table 1. Examples of the sentiment score comparison between an original tweet and its translated version via SentiStrength

| Original Korean Tweet | | Translated Tweet | |
| --- | --- | --- | --- |
|  | |  | |
| “모더나 2차 접종 후 하루 뒤, 몸살 같은 증세에 하루 종일 누워 있다 이제 좀 정신을 차렸다. 한달 간격으로 맞은 같은 약이 이렇게 다른 종류의 반응을 가져온다는게 신기하고 놀랍네.” | | “One day after the second dose of Moderna, I was lying in bed all day with symptoms like body aches. Now I came to my senses.” | |
| Positive: 1 | Negative: -1 | Positive: 2 | Negative: -4 |
| “3차 화이자 접종 다섯시간경과. 1차, 2차 아스트라제네카보다 접종 부위가 10배는 더 아픔.” | | “Five hours after the 3rd Pfizer inoculation, the injection site hurt 10 times more than the 1st, 2nd AstraZeneca.” | |
| Positive: 1 | Negative: -1 | Positive: 1 | Negative: -3 |
| “1차 아스트라제네카 2차 화이자 3차 화이자였는데, 2차때가 젤 아팠던것 같아요. 며칠 심장쪽이 따끔따끔하기도 했었거든요. 3차는 겨드랑이쪽만 부었었는데, 며칠 지난 지금은 가라앉았어요.” | | “1st AstraZeneca 2nd Pfizer 3rd Pfizer I think the 2nd time was the most painful I had a tingling in my heart for a few days.” | |
| Positive: 1 | Negative: -1 | Positive: 1 | Negative: -4 |

One might wonder if it was impossible to utilize a sentiment analysis program to support Korean, such as KNU Korean Sentiment Dictionary (<http://dilab.kunsan.ac.kr/knu/knu.html>). However, that program revealed several serious flaws. First, because KNU Korean Sentiment Dictionary used words rather than sentences as basic units, it simply determined whether a word was positive or negative. Hence, KNU Korean Sentiment Dictionary exposed a critical drawback that could not precisely capture a subtle nuance in Korean sentences. Second, the KNU Korean Sentiment Dictionary contained only a limited set of words. More importantly, it did not include quite a few critical terms extracted from our dataset, such as headache, muscle pain, cold, or sickness. We could not use the Korean sentiment analysis program due to these constraints.
