# Supplementary material for: A Comprehensive Analysis of COVID-19 Vaccine Discourse by Vaccine Brand on Twitter in Korea: Topic and Sentiment Analysis
Source: J Med Internet Res. 2023 Jan 31;25:e42623. doi: 10.2196/42623 (PMC9891356; doi:10.2196/42623)
Supplement: Multimedia Appendix 6 [file jmir_v25i1e42623_app6.docx]

 Multimedia Appendix 6. The results of autoregression between sentiment score and time by Covid-19 vaccine brands.

| Vaccine brand | Parameter Estimate | Standard Error | *p*-value |
| --- | --- | --- | --- |
| Topic #0 Pfizer | 0.000188 | 0.000181 | 0.3053 |
| Topic #1 Moderna | 0.000095 | 0.000123 | 0.4405 |
| Topic #2 AstraZeneca | 0.000044 | 0.000133 | 0.7401 |
| Topic #3 Janssen | 0.000200 | 0.000173 | 0.2508 |
| Topic #4 Novavax | 0.000051 | 0.000163 | 0.7548 |
| Topic #5 Unspecified | 0.000253 | 0.000350 | 0.4736 |
